# Characterization and pathogenicity of multidrug-resistant coagulase-negative *Staphylococci* isolates in chickens

**DOI:** 10.1007/s10123-023-00354-0

**Published:** 2023-04-13

**Authors:** Hend K. Sorour, Azhar G. Shalaby, Marwa A. Abdelmagid, Reham A. Hosny

**Affiliations:** Reference Laboratory for Veterinary Quality Control on Poultry Production, Animal Health Research Institute, Agricultural Research Center, Giza, Egypt

**Keywords:** Coagulase negative, Enterotoxin, Mec*A*, Resistance, *Staphylococci* species

## Abstract

The pathogenic potential of vancomycin and methicillin-resistant coagulase-negative *Staphylococci* (VMRCoNS) on Egyptian poultry farms has received little attention. Therefore, this study aims to study the prevalence of CoNS in imported poultry flocks and commercial poultry farms, evaluate the presence of virulence and antibiotic resistance genes (*sea*, *seb*, *sec*, *sed*, *see*, and *mecA*), and assess their pathogenicity in broiler chicks. Seven species were identified among 25 isolates, such as 8 *S. gallinarum*, 5 *S. saprophyticus*, 5 *S. chromogens*, 3 *S. warneri*, 2 *S. hominis*, 1 *S. caprae*, and 1 *S. epidermidis*. All isolates were resistant to clindamycin, doxycycline, vancomycin, methicillin, rifampicin, and penicillin. The *mecA* gene was confirmed in 14 isolates, while the *sed* gene was revealed in seven isolates. Commercial 1-day-old Ross broiler chicks were divided into eight groups of three replicates (10 birds/group): group Ӏ was negative control; groups (П, Ш, IV, V, VI, VII, and VIII) were subcutaneously inoculated with 10^8^ CFUml^−1^ of *S. hominis*, *S. caprae*, *S. epidermidis*, *S. gallinarum*, *S. chromogens*, *S. warneri*, and *S. saprophyticus*, respectively. Groups VIII and V had mortality rates of 100% and 20%, respectively, with no evidence of mortalities in the other groups. The highest re-isolation of CoNS species was recorded in groups VII, VIII, and V. Postmortem and histopathological examination revealed the common presence of polyserositis in the internal organs, and hepatic and myocardial necrosis in groups IV, V, and VI. These findings revealed the pathogenic potential of CoNS, so special attention must be directed toward their public health impact.

**Supplementary Information:**

The online version contains supplementary material available at 10.1007/s10123-023-00354-0.

## Introduction

Staphylococcal infections in poultry represent one of the major economic problems, which are linked to lameness and its consequences on production parameters, mortality, and condemnation of carcasses at slaughterhouses (Szafraniec et al. 2020).

*Staphylococci* species are saprophytic gram-positive, facultative anaerobe, non-motile, and non-sporing spherical bacteria that can be found in air, litter, feed, and water. They colonize the skin and mucous membranes of humans and animals (Čuvalová et al. 2015). They are divided into two groups according to their ability to coagulate blood plasma: the coagulase-positive (CoPS) and coagulase-negative *Staphylococci* (CoNS) (Čuvalová et al. 2015; Pyzik et al. 2019). The coagulase-positive *Staphylococcus aureus* (*S. aureus*) appears to be the most important species associated with food poisoning outbreaks that are mainly caused by ingestion of food contaminated with enterotoxins (Pyzik et al. 2019). *Staphylococcus aureus* may produce different classical enterotoxins, such as A, B, C, D, and E, that are resistant to heat, pH, and pepsin digestion (Argudín et al. 2010). Other *Staphylococcus* species termed coagulase-negative *Staphylococci* (CoNS) have gained importance over the past decade, as they have been involved in different infections in humans and animals (Balaban and Rasooly 2000; Čuvalová et al. 2015; Pyzik et al. 2019). The most common CoNS associated with poultry infections are *S. lentus*, *S. xylosus*, *S. cohnii*, and *S. hyicus* (Stępień-Pyśniak et al. 2017). CoNS have frequently been isolated from chicken nares and skin, including *S. chromogenes*, *S. gallinarum*, *S. xylosus*, and *S. epidermidis* (Pyzik et al. 2019; von Eiff et al. 2002). The pathogenic potential of CoNS species depends on the production of enterotoxin genes which could be transmitted by coexistence with pathogenic *Staphylococci* (Osman et al. 2020). Two available studies in Egypt and Poland have reported the detection of some enterotoxins, such as sec, see, and seb, in some CoNS species in poultry (El-Nagar et al. 2017; Pyzik et al. 2019). It has been reported that experimental infection of 10-day-old broiler chicks with *S. xylosus*, *S. sciuri*, and *S. lentus* led to mild subclinical disease with histopathological lesions in the liver, spleen, and intestine (Shokry et al. 2018).

The extensive use of antibiotics in the veterinary sector results in their persistent release into the environment and the emergence of antibiotic-resistant bacteria (Manyi-Loh et al. 2018). It was reported that CoNS had increased the rates of antibiotic resistance, even higher than *S. aureus*, which reduces the available therapeutic options (Silva et al. 2022). There are numerous reports of nosocomial infections caused by methicillin and vancomycin-resistant CoNS (Al-Tamimi et al. 2020; Cercenado 2010; Srinivasan et al. 2002). Previous studies revealed the isolation of methicillin-resistant CoNS from commercial chickens in Belgium, Egypt, and Portugal in percentages of 12.5%, 51.7%, and 19.6, respectively (El-Nagar et al. 2017; Nemeghaire et al. 2014; Silva et al. 2022). Bhargava and Zhang (2014) reported the contamination of chicken meats obtained from retail stores in the USA with 7.9% methicillin-resistant CoNS. Until now, there are no available studies concerning the isolation of vancomycin-resistant CoNS from commercial poultry farms in Egypt. Osman et al. (2016) reported the isolation of vancomycin-resistant CoNS from the Egyptian poultry meats collected from retail supermarkets in a percentage of 27.8%.

Although there has been an increase in clinical research interest in CoNS recently, there is little data on their prevalence, antibiotic resistance profiles, and pathogenicity on Egyptian poultry farms. Furthermore, the information is even more limited regarding vancomycin and methicillin-resistant CoNS. Therefore, this study aims to identify species of CoNS isolated from different types of poultry samples, to assess the susceptibility of isolates to different antibiotics, particularly vancomycin and methicillin, to detect the presence of enterotoxins and methicillin resistance genes (*mecA*), and to evaluate their pathogenicity in one-day broiler chicks.

## Materials and methods

### Samples

Between January 2022 and April 2022, 1020 pooled organ samples (trachea-lung, liver-heart, and intestine) were collected from imported healthy chicks, ducklings, and turkey poults. Furthermore, a total of 1090 pooled organ samples (trachea-lung, liver-heart, and intestine) were collected from healthy 1-day-old broiler chicks, broiler breeders, and broilers from nine governorates (Cairo, Dakahlia, Giza, Behera, Menofia, Alexandria, Sharkia, Qalyubia, and Minia) (Table 1). Each ten pooled samples constituted one sample. The broiler breeders ranged in age from 196 to 357 days, while the age of broilers ranged from 3 to 38 days. The samples were transported in sterile plastic bags under aseptic conditions to the Reference Laboratory for Veterinary Quality Control on Poultry Production, Dokki, Egypt (RLQP), in cool boxes with ice packs to isolate CoNS.

**Table 1:** Prevalence of positive samples for CoNS species recovered from commercial poultry farms in different governorates

Governorate	Species	Number of examined samples	Number (%) of positive samples for CoNS species
Cairo	One-day-old chicks	30	20 (66.7%)
	Broiler breeders	20	0
	Broilers	0	0
Total		50	20 (40%)
Dakahlia	One-day-old chicks	100	0
	Broiler breeders	20	10 (50%)
	Broilers	60	20 (33.3%)
Total		180	30 (16.7%)
Giza	One-day-old chicks	160	0
	Broiler breeders	0	0
	Broilers	220	110 (50%)
Total		380	110 (29%)
Behera	One-day-old chicks	40	0
	Broiler breeders	20	0
	Broilers	40	0
Total		100	0
Menofia	One-day-old chicks	40	0
	Broiler breeders	0	0
	Broilers	40	0
Total		80	0
Alexandria	One-day-old chicks	50	20 (40%)
	Broiler breeders	20	10 (50%)
	Broilers	40	0
Total		110	30 (27.3%)
Sharkia	One-day-old chicks	40	0
	Broiler breeders	0	0
	Broilers	40	10 (25%)
Total		80	10 (12.5%)
Qaliobia	One-day-old chicks	0	0
	Broiler breeders	0	0
	Broilers	40	10
Total		40	10 (25%)
Minia	One-day-old chicks	30	10 (33.3%)
	Broiler breeders	0	0
	Broilers	40	0
Total		70	10 (14.3%)

The percentage of positive samples for CoNS species in each governorate was calculated according to corresponding number of examined samples

### Isolation and identification of CoNS

CoNS were isolated according to El-Nagar et al. (2017) and Shokry et al. (2018) by inoculating 10 g of samples into 90 ml of buffer peptone water (Oxoid Limited, Thermo Fisher Scientific Inc., UK) and incubating at 37 °C for 24 h. A loopful of enriched samples was then streaked on Baird Parker agar (Oxoid Limited, Thermo Fisher Scientific Inc., UK) and Mannitol Salt agar plates (Oxoid Limited, Thermo Fisher Scientific Inc., UK), and incubated at 37 °C for 48 h. The plates were examined for the presence of black colonies without clear or opaque zones on Barid Parker and yellow or red colonies on Mannitol Salt agar. The suspected CoNS colonies were identified according to Holt et al. (1994) using different biochemical tests such as coagulase, catalase, sugars fermentation (mannose, sucrose, mannitol, trehalose, maltose, lactose, and raffinose), urea, Voges-Proskauer, ONPG, and novobiocin sensitivity (Table S1).

### Phenotypic antimicrobial susceptibility test

The phenotypic antimicrobial profile of the isolated CoNS was assessed by disk diffusion technique using 12 commercial disks (Oxoid Limited, Thermo Fisher Scientific Inc., UK) following Clinical and Laboratory Standard Institute guidelines (CLSI 2020). Tobramycin and methicillin breakpoints for the disk diffusion test were interpreted according to CLSI (2017). The antibiotic disks and corresponding concentrations used in this study were ofloxacin (5 μg), levofloxacin (5 μg), clindamycin (2 μg), doxycycline (30 μg), cefoxitin (30 μg), methicillin (5 μg), rifampicin (5 μg), tetracycline (30 μg), trimethoprim-sulphamethoxazole (25 μg), penicillin (10 μg), tobramycin (10 μg), and novobiocin (5 μg).

### Determination of minimum inhibitory concentration (MIC) for vancomycin using the broth macro-dilution method

The MIC test was used to determine the susceptibility of CoNS isolates to vancomycin as the disk diffusion test cannot distinguish between vancomycin-susceptible, -intermediate, and -resistant isolates. All of these give inhibition zones with similar diameter sizes (CLSI 2020). The macro-dilution broth technique was used to determine the minimum inhibitory concentration of vancomycin against 25 isolated CoNS following the Clinical and Laboratory Standards Institute (CLSI 2017; CLSIM07-A9, 2012). Vancomycin was obtained from Sigma-Aldrich (St. Louis, USA). The MIC breakpoints of vancomycin against *Staphylococcus* species range from 4 to 32 μg ml^−1^ (CLSI 2020). To obtain the different concentrations of vancomycin, a twofold serial dilution was carried out from the working solution (1 mg ml^−1^). For the preparation of bacterial inoculums, pure overnight yellow or red colonies cultured on mannitol salt agar medium were suspended in Mueller–Hinton broth (MHB) medium (Oxoid Limited, Thermo Fisher Scientific Inc., UK) and adjusted to 5 × 10^5^ CFUml^−1^ as described by CLSIM07-A9 (2012). In the dilution series, 100 µl of each bacterial inoculum was added to 2 ml of MHB medium containing vancomycin. The tubes were incubated for 16 to 20 h at 35 ± 2 °C. The last two tubes contained positive control for each *Staphylococcus* species isolate and negative control for MHB. The MIC was considered the lowest vancomycin concentration, which produced no visible growth (no turbidity recorded).

### Screening for 16s rRNA, sea, seb, sec, sed, see, and mecA genes in CoNS isolates using a uniplex and multiplex polymerase chain reaction

The DNAs of 25 CoNS isolates were extracted using a QIAamp DNA Mini kit (Qiagen, Germany, GmbH) according to the manufacturer’s protocol. The primer sequences and amplicon sizes used to detect *16 s rRNA*, four toxin genes (*sea*, *seb*, *sec*, *sed*, *see*), and a methicillin-resistant gene (*mecA*) were outlined in Table S2. The PCR amplification was performed in a Thermal Cycler (Applied biosystem 2720). The strains used as positive controls for *seb*, *sec*, *see*, and *mecA* genes were obtained from ISO 17025 accredited biotechnology unit, RLQP, Egypt (El-Nagar et al. 2017). Furthermore, *S. aureus* FRI151m and *S. aureus* FRI913 were used as positive controls for *sed* and *sea* genes, respectively, while *Escherichia coli* strain (NCIMB 50,034) was used as a negative control.

Multiplex PCR was conducted using primers specific for the *sea*, *seb*, *sec*, *sed*, and *see* genes in a final reaction volume of 50 μl including 25 μl of EmeraldAmp Max PCR Master Mix (Takara, Japan), 11 μl of sterile distilled water, 1 μl of each primer, and 6 μl of DNA extract. The cycling conditions included an initial denaturation at 94 °C for 5 min, followed by 35 cycles of denaturation for 30 s at 94 °C, annealing for 45 s at 50 °C, extension for 45 s at 72 °C, and final extension for 10 min at 72 °C (Mehrotra et al. 2000). Two uniplex PCR were performed using primers specific for the *16S rRNA* and *mecA* genes in a final reaction volume of 25 μl containing 12.5 μl of EmeraldAmp Max PCR Master Mix (Takara, Japan), 5.5 μl of sterile distilled water, 1 μl of each primer, and 5 μl of DNA extract. The amplification conditions were 5 min of initial denaturation at 94 °C, followed by 35 cycles of denaturation at 94 °C for 30 s, annealing at 55 °C for 40 s, extension at 72 °C for 45 s, and final extension at 72 °C for 10 min (Mason et al. 2001; McClure et al. 2006). Then, the amplified products were separated in 1.5% agarose (AppliChem, Germany, GmbH) in a 1 × Tris/Borate/EDTA buffer (TBE) at 5 Vcm^−1^. PCR products were visualized under ultraviolet illumination.

### Pathogenicity of CoNS in 1-day-old chicks

#### Bacterial inoculums

A total of seven isolates, *S. hominis*1, *S. caprae*, *S. epidermidis*, *S. gallinarum* 6, *S. chromogens* 5, *S. warneri* 3, and *S. saprophyticus* 3, were selected based on the molecular results (Table S3). All selected isolates were resistant to clindamycin, doxycycline, vancomycin, methicillin, rifampicin, and penicillin and displayed positive amplification for the *sed* and *mecA* genes except *S. epidermidis*, which contained only *mecA* gene (Table S3)*.*

#### Experimental design

A total of 240 commercial 1-day-old Ross broilers are purchased from a commercial hatchery in Behera. Chicks were housed in battery cages located in a biosafety level 2 experimental room in RlQP. Temperature, lighting, and relative humidity were adjusted according to Ross broiler management specifications (Aviagen 2018). Birds were provided on-demand water and a starter diet following Ross 308 broiler nutrition specifications guidelines Aviagen, (2014). Tracheal, cloacal, and environmental swabs were taken from the chicks (extra than 240) and cages and walls of the experimental room, respectively before the housing of chicks and examined for staphylococcal infection using PCR for the *16srRNA* gene.

The design of the experimental study was conducted as described by Shokry et al. (2018), with some modifications in the inoculated species and age. Chicks were divided into eight groups of three replicates (10 birds/group) as follows: group Ӏ was negative control, groups (П, Ш IV, V, VI, VII, and VIII) were subcutaneously inoculated in the bottom of neck region with 0.5 ml containing 10^8^ CFUml^−1^ of *S. hominis*, *S. caprae*, *S. epidermidis*, *S. gallinarum*, *S. chromogens*, *S. warneri*, and *S. saprophyticus*, respectively. Each group was housed in separate three-level wire netted-floor batteries with two individual cages, each holding five chicks. The floors of the cages were covered with daily-replaced papers. The clinical signs and mortality were recorded daily. The birds were euthanized on day 10 for re-isolation of inoculated CoNS and examination of gross and histopathological lesions.

#### CoNS re-isolation

Heart, liver, intestine, and yolk samples were examined for CoNS according to El-Nagar et al. (2017) and Shokry et al. (2018) on Baird-Parker and mannitol salt agar plates as previously described. The re-isolated CoNS were further identified using different biochemical tests according to Holt et al. (1994) and screened for antibiotic resistance, *sed*, and *mecA* genes to confirm their identity to the CoNS strains used in the challenge, as previously described.

#### Histopathological examination

Tissue samples were collected from the heart, liver, and intestine of euthanized birds. These samples were fixed immediately in neutral buffered formalin 10%, processed, and stained with hematoxylin and eosin stain (H&E stain), as stated by Suvarna et al. (2018). The histopathology scoring was as follows: 0 = no lesions, 1 = mild lesions, 2 = moderate lesions, and 3 = severe lesions, according to Gibson-Corley et al. (2013).

## Results and discussion

### Isolation and identification of CoNS species

CoNS were isolated in 250 pooled samples out of 2110 from the different types of samples (imported chicks, imported poults, 1-day-old chicks, broiler breeder, and broilers) in percentages of 3.2%, 6.7%, 10.2%, 25%, and 28.81%, respectively, representing 25 isolates. The isolation of CoNS in this study was at a prevalence of 11.8%, which was consistent with those reported in a previous study in Egypt by El-Nagar et al. (2017) that reported a prevalence of 19.3% and lower than those in previous studies in Ghana, Poland, Brazil, and Portugal that reported a prevalence of 70%, 53%, 36.4%, and 26%, respectively (Boamah et al. 2017; Pimenta et al. 2021; Pyzik et al. 2019; Silva et al. 2022). The prevalence of the *Staphylococci* species in poultry varied widely and might be attributed to the differences in the geography and sources of samples (Silva et al. 2022).

The biochemical analysis of the suspected CoNS colonies revealed that the identified isolates were 8 *S. gallinarum*, 5 *S. saprophyticus*, 5 *S. chromogens*, 3 *S. warneri*, 2 *S. hominis*, *S. caprae*, and *S. epidermidis.* All isolated CoNS species did not ferment mannitol and grew as small red colonies except eight isolates of *S. gallinarum* that fermented mannitol and produced yellow colonies. In a report by El-Nagar et al. (2017), six CoNS species, including S. *warneri*, *S. epidermidis*, *S. saprophyticus*, and others, were recovered from poultry samples collected from the chicken production cycle in the Luxor governorate. Pyzik et al. (2019) detected 22 CoNS species in Poland’s diseased broiler chicken and turkey samples, including *S. saprophyticus*, *S. epidermidis*, *S. chromogenes*, *S. hominis*, *S. warneri*, and others. A study in Thailand reported isolating nine CoNS species, including *S. gallinarum*, *S. saprophyticus*, and others, from broiler feces (Rueanghiran et al. 2017). Boamah et al. (2017) revealed the isolation of six CoNS species, including *S. saprophyticus* and others, from poultry litters in Ghana.

**Fig. 1:**
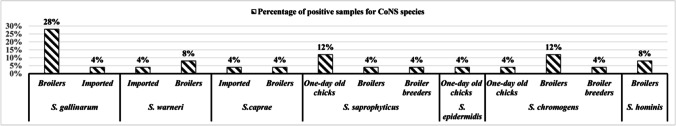
Distribution of the isolated CoNS species in imported and local poultry samples. A clustered bar chart illustrating the percentage of positive samples for CoNS species in different poultry samples, including imported, 1-day-old chicks, broilers, and broiler breeders

*Staphylococcus gallinarum* was the main species isolated in this study with a prevalence of 32%, which was consistent with those reported in a study by Rueanghiran et al. (2017) in Thailand that reported a prevalence of 25% (Fig. 1). *Staphylococcus warneri* and *S. hominis* were isolated at a prevalence of 12% and 8% which was lower than those reported in a previous study in Egypt (El-Nagar et al. 2017) that reported a prevalence of 17.3%, 10.3%, and higher than those reported in a study by Pyzik et al. (2019) in Poland that reported a prevalence of 0.8% and 1.7% (Fig. 1). *Staphylococcus epidermidis* was isolated at a prevalence of 4% which was lower than those reported in previous studies in Egypt and Poland that reported a prevalence of 10.3% and 7.2%, respectively (El-Nagar et al. 2017; Pyzik et al. 2019) (Fig. 1). *Staphylococcus saprophyticus* was isolated at a prevalence of 20% which was higher than those reported in previous studies in Ghana, Egypt, Thailand, and Poland that reported a prevalence of 2%, 10.3%, 10%, and 8.4%, respectively (Boamah et al. 2017; El-Nagar et al. 2017; Pyzik et al. 2019; Rueanghiran et al. 2017) (Fig. 1). *Staphylococcus chromogenes* was isolated at a prevalence of 20%, which was higher than those reported in a study by Pyzik et al. (2019) in Poland, which reported a prevalence of 3.4%. Furthermore, this study reported the first isolation of *S. caprae* from imported poultry samples with a prevalence of 4% (Fig. 1).

The highest incidence of CoNS isolates was recorded in the Giza governorate, with a percentage of 50% (110/220). In contrast, the lowest incidence was recorded in Sharkia, Qalyubia, and Minia governorates, with a percentage of 4.5% (10/220) each. Furthermore, there were no reported positive CoNS isolates in Behera and Menofia governorates (Fig. S1).

The present study revealed that *CoNS* isolates were mainly recovered from the liver and heart in a percentage of 52% (130/250), followed by trachea-lung and intestine in a percentage of 24% (60/250) for each*.* It was found that *S. chromogens*, *S. saprophyticus*, and *S. gallinarum* were recovered from all organs, while *S. warneri* was recovered from liver-heart and trachea-lung. On the other hand, *S. hominis*, *S. caprae*, and *S. epidermidis* were mainly recovered from liver-heart (Fig. S2).

### Antimicrobial susceptibility testing of isolated CoNS isolates using disk diffusion and broth macro-dilution methods

Antimicrobial susceptibility testing of CoNS isolates revealed 100% resistance to clindamycin, doxycycline, methicillin, rifampicin, and penicillin, with high susceptibility to ofloxacin and levofloxacin in percentages of 76% and 68%, respectively (Table 2). Resistance to novobiocin and cefoxitin was confirmed in 52% and 56%, respectively, in the tested isolates (Table 2). The MIC results of vancomycin did not display inhibition of the growth of tested isolates. Vancomycin had high MIC values of 64 μg ml^−1^ against all tested CoNS isolates except *S. caprae* and *S. epidermidis*, which exhibited MIC values of 32 μg ml^−1^. The resistance to vancomycin and doxycycline observed in this study contrasts with the findings of Rueanghiran et al. (2017), who reported 100% susceptibility to vancomycin and 43.75% resistance to doxycycline. Osman et al. (2016) study revealed the isolation of vancomycin- and methicillin-resistant CoNS from poultry meat (breast and thigh) in percentages of 27.8% and 52.8%, respectively. To the best of our knowledge, this is the first study to report the isolation of vancomycin- and methicillin-resistant CoNS from different local and imported poultry samples. Contrary to the findings of Pyzik et al. (2019); Rueanghiran et al. (2017); and Silva et al. (2022), who reported resistance to clindamycin in a percentage of 45%, 5.5%, and 97.3%, respectively, the level of resistance in this study was higher. The resistance to penicillin in this study was higher than those obtained in previous investigations (Boamah et al. 2017; Pyzik et al. 2019; Silva et al. 2022), which revealed resistance in a percentage of 28.5%, 54.3%, and 65.3%, respectively. It was reported that the tetracycline resistance in this study was 96%, higher than those obtained by Boamah et al. (2017); Pyzik et al. (2019); Rueanghiran et al. (2017); and Silva et al. (2022), who reported resistance in a percentage of 35%, 57.03%, and 59.1% and 85.3%, respectively.

**Table 2:** Antimicrobial susceptibility of the isolated CoNS using the disk diffusion test

Antibiotic	Resistant isolates number (%)	Resistance breakpoints for zones of disk diffusion	Intermediate isolates number (%)	Intermediate breakpoints for zones of disk diffusion	Sensitive isolates number (%)	Sensitive breakpoints For zones of disk diffusion
Ofloxacin (OF)	5 (20%)	≤ 14	1 (4%)	15–17	19 (76%)	≥ 18
Cefoxitin (FOX)	14 (56%)	≤ 24	0		11 (44%)	≥ 25
Levofloxacin (Lev)	6 (24%)	≤ 15	2 (8%)	16–18	17 (68%)	≥ 19
Clindamycin (CD)	25 (100%)	≤ 14	0	15–20	0	≥ 21
Doxycycline (DO)	25 (100%)	≤ 12	0	13–15	0	≥ 16
Methicillin (Met)	25 (100%)	≤17	0		0	≥22
Rifampicin (RIF)	25 (100%)	≤ 16	0	17–19	0	≥ 20
Tetracycline (TET)	24 (96%)	≤ 14	1 (4%)	15–18	0	≥ 19
Trimethoprim-sulphamethoxazole (SXT)	21 (84%)	≤ 10	0	11–15	4 (16%)	≥ 16
Penicillin (P)	25 (100%)	≤ 28	0		0	≥ 29
Tobramycin (TOB)	11 (44%)	≤ 12	2 (8%)	13–14	12 (48%)	≥ 15
Novobiocin (NV)	13 (52%)	≤ 17	0	18–21	12 (48%)	22

The percentage of sensitive, intermediate, and resistant CoNS isolates were calculated according to total number of post isolates for CoNS (25), breakpoints for disk diffusion were according to CLSI 2017 and 2020

It was found that cefoxitin is used as a delegate for *mecA*-mediated oxacillin resistance (CLSI 2017). Considering this study, 14 isolates (56%) were oxacillin and methicillin-resistant CoNS at the phenotypic susceptibility analysis, presenting resistance to cefoxitin which was higher than those obtained by Boamah et al. (2017); Pyzik et al. (2019); and Silva et al. (2022), who reported resistance percentages of 16%, 16,5%, and 38.7%, respectively.

Multi-resistance pattern to the 12 antimicrobials tested was displayed in all 25 CoNS isolates (Table 3)*.* Two isolates (*S. saprophyticus* and *S. gallinarum*) showed 100% (12/12) resistance to the tested antibiotics, while four isolates (*S. chromogens*, *S. caprae*, *S. epidermidis*, and *S. saprophyticus*) exhibited 91.7% (11/12) resistance to them (Table 3). Only one isolate of *S. gallinarum* showed resistance to 83.3% (10/12) of the tested antibiotics, while eight isolates (*3 S. gallinarum*, 2 *S. chromogens*, *2 S. saprophyticus*, and *S. warneri*) exhibited 75% (9/12) resistance to them (Table 3).

**Table 3:** Phenotypic and genotypic characterization of the isolated CoNS species

Isolates	Phenotypic antibiotic resistance pattern	Genotypic
*S. hominis* 1	CD, DO, MET, RIF, TE, P, SXT	*16srRNA*, *sed*, *mecA*
*S. hominis* 2	FOX, CD, DO, MET, RIF, TE, P, SXT	*16srRNA*, *mecA*
*S. chromogens* 1	OF, FOX, Lev, CD, DO, Met, RIF, TE, SXT, P, TOB	*16srRNA*
*S. chromogens* 2	CD, DO, Met, RIF, TE, SXT, P, TOB	*16srRNA*, *mecA*
*S. chromogens* 3	FOX, CD, DO, Met, RIF, TE, SXT, P, TOB	*16srRNA*
*S. chromogens* 4	Lev, CD, DO, Met, RIF, TE, SXT, P, TOB	*16srRNA*, *mecA*
*S. chromogens* 5	CD, DO, Met, RIF, TE, P, TOB	*16srRNA*, *sed*, *mecA*
*S. warneri* 1	CD, DO, Met, RIF, TE, P	*16srRNA*, *mecA*
*S. warneri* 2	FOX, CD, DO, Met, RIF, TE, P	*16srRNA*, *mecA*
*S. warneri* 3	FOX, Lev, CD, DO, Met, RIF, TE, SXT, P	*16srRNA*, *sed*, *mecA*
*S. caprae*	OF, FOX, Lev, CD, DO, Met, RIF, TE, SXT, P, TOB	*16srRNA*, *sed*, *mecA*
*S. epidermidis*	OF, FOX, Lev, CD, DO, Met, RIF, TE, SXT, P, TOB	*16srRNA*, *mecA*
*S. saprophticus* 1	CD, DO, Met, RIF, TE, SXT, P, TOB, NV	*16srRNA*
*S. saprophticus* 2	OF, FOX, Lev, CD, DO, Met, RIF, TE, SXT, P, NV	*16srRNA*
*S. saprophticus* 3	OF, FOX, Lev, CD, DO, Met, RIF, TE, SXT, P, TOB, NV	*16srRNA*, *sed*, *mecA*
*S. saprophticus* 4	FOX, CD, Met, RIF, TE, SXT, P, NV	*16srRNA*
*S. saprophticus* 5	CD, DO, Met, RIF, TE, SXT, P, TOB, NV	*16srRNA*, *mecA*
*S. gallinarum* 1	FOX, CD, DO, Met, RIF TE, SXT, P, NV	*16srRNA*, *mecA*
*S. gallinarum* 2	FOX, CD, DO, Met, RIF, TE, SXT, P, NV	*16srRNA*
*S. gallinarum* 3	CD, DO, Met, RIF, TE, SXT, P, NV	*16srRNA*
*S. gallinarum* 4	CD, DO, Met, RIF, TE, P, NV	*16srRNA*
*S. gallinarum* 5	CD, DO, Met, RIF, TE, SXT, P, NV	*16srRNA*
*S. gallinarum* 6	OF, FOX, Lev, CD, DO, Met, RIF, TE, SXT, P, TOB, NV	*16srRNA*, *sed*, *mecA*
*S. gallinarum* 7	FOX, CD, DO, Met, RIF, TE, SXT, P, TOB, NV	*16srRNA*
*S. gallinarum* 8	CD, DO, Met, RIF, TE, SXT, P, TOB, NV	*16srRNA*, *sed*

*OF* ofloxacin, *FOX* cefoxitin, *Lev* levofloxacin, *CD* clindamycin, *DO* doxycycline, *Met* methicillin, *RIF* rifampicin, *TE* tetracycline, *SXT* trimethoprim-sulphamethoxazole, *P* penicillin, *TOB* tobramycin, and *NV* novobiocin

### Screening for 16srRNA, sea, seb, sec, sed, see, and mecA genes in CoNS isolates using a conventional polymerase chain reaction

All CoNS isolates revealed a positive amplification for the *16srRNA* gene (*Staphylococcus* genus specific) at 791 bp. Similarly, Vadari et al. (2006) confirmed the isolation of *Staphylococcus* species from poultry litter samples using the *16srRNA* gene.

Staphylococcal enterotoxin genes were examined in all CoNS isolates to assess their enterotoxigenicity. The results revealed the detection of the *sed* gene (278 bp) in 28% (7/25) of isolates, including *S. hominis*, *S. chromogens*, *S. warneri*, *S. caprae*, *S. saprophyticus*, and two isolates of *S. gallinarum* (Table 3)*.* Other enterotoxin genes such as *sea*, *seb*, *sec*, and *see* were not detected (Table 3)*.* On the other hand, El-Nagar et al. (2017) reported the detection of the *sec* gene in one isolate of *S. simulans* and two isolates of *S. xylosus*, whereas the *see* gene was detected in one isolate of *S. warneri*, *S. xylosus*, and *S. simulans* in a percentage of 10.3% for each gene. Pyzik et al. (2019) reported the detection of the *see* gene in a percentage of 1.6% in two isolates of *S. hominis*, while the *seb* gene was detected in a percentage of 0.8% in *S. epidermidis.*

Methicillin resistance was confirmed by detecting the *mecA* gene, which codes for an alternative binding protein (PBP2a) with a low affinity for beta-lactam antibiotics. The *mecA* gene detection method is considered the gold standard for identifying methicillin-resistant isolates. In this study, *mecA* gene (310 bp) was revealed in 56% (14/25) of isolates, which was higher than reported in the previous study by Osman et al. (2016) that revealed its detection in CoNS isolates recovered from poultry meat in a percentage of 33.3% and lower than obtained by (Silva et al. 2022) that revealed its detection in 100% of CoNS isolates recovered from commercial chickens. All 14 *mecA* positive CoNS isolates were phenotypically methicillin resistant (FOX) and this contrast with the findings of Silva et al. (2022) that revealed the detection of the *mecA* gene in 46 cefoxitin-susceptible CoNS isolates. *mecA* gene was detected in the present study in two isolates of *S. hominis*, *S. gallinarum*, and *S. saprophyticus*; three isolates of *S. warneri* and *S. chromogens*; and one isolate of *S. epidermidis* and *S. caprae*. In contrast, Osman et al. (2016) revealed the detection of the *mecA* gene in four isolates of *S. epidermidis*, two isolates of *S. hominis*, and others. It was unexpectedly reported in this investigation that the *mec*A gene was not detected in 11 phenotypically methicillin-resistant CoNS (MET disk); this may be related to the presence of a different *mec* gene (*mecC*) (Cikman et al. 2019). Osman et al. (2016) reported similar results regarding the absence of the *mec*A gene in seven phenotypically methicillin-resistant CoNS isolates recovered from poultry meat in Egypt. Although *mecC*-containing MRCoNS strains are not common in the veterinary sector, they could still pose a threat since phenotypic testing is insufficient to detect this gene.

### Pathogenicity of CoNS species in 1-day-old chicks

The pathogenicity of seven selected CoNS isolates, including *S. hominis*1, *S. caprae*, *S. epidermidis*, *S. gallinarum* 6, *S. chromogens* 5, *S. warneri* 3, and *S. saprophyticus* 3, was evaluated in 1-day-old chicks. The selection of the inoculation dose (10^8^) used in the experimental study was based on a study by Shokry et al. (2018) that revealed the absence of mortalities in 10-day-old broiler chicks subcutaneously inoculated with *S. xylosus*, *S. sciuri*, and *S. lentus*.

All environmental swabs taken from the cages and walls of the experimental room before the housing of chicks were negative for CoNS infection.

### Clinical signs, mortality, and gross lesions

The negative control group (Ӏ) showed no signs. The clinical signs observed in all challenged groups (П, Ш IV, V, VI, VII, and VIII) were mild, including depression and ruffled feathers. Groups VIII and V challenged with *S. saprophyticus* and *S. gallinarum* had mortality rates of 100% and 20%, respectively, compared to the other challenged groups (П, Ш, IV, V, VI, and VII) that revealed no evidence of mortalities (Table S4). These observed mortalities suggest the severity of these isolates to cause death, which showed resistance to all tested antimicrobials in the antibiogram assay (Table S3). All mortalities in group VIII were observed on days 2 and 3 post-infection at percentages of 80% and 20%, respectively, whereas group V had a mortality rate of 20% on day 4 post-infection (Table S4).

The negative control group (Ӏ) showed no gross lesions. All challenged groups (П, Ш, IV, V, VI, VII, and VIII) showed polyserositis in their internal organs. In particular, groups Ш and VIII challenged with *S. caprae*, and *S. saprophyticus* exhibited polyserositis, yolk sac retention, yolk sacculitis, and airsacculitis. Groups V and VI, challenged with *S. gallinarum* and *S. chromogens*, exhibited polyserositis, unabsorbed yolk, and necrotic heart and liver. Groups IV and VII challenged with *S. epidermidis* and *S. warneri* exhibited polyserositis and necrotic liver. The necrotic heart was observed only in groups IV, V, and VI challenged with *S. epidermidis*, *S. gallinarum*, and *S. chromogens*, whereas necrotic liver was observed only in groups IV, V, VI, VII, and VIII challenged with *S. epidermidis*, *S. gallinarum*, *S. chromogens*, *S. warneri*, and *S. saprophyticus* (Table S4). Despite the absence of necrotic heart lesions in group VIII challenged with *S. saprophyticus*, 100% deaths were recorded. It might be attributed to the massive cytokine production and toxic shock syndrome (TSS), which may have resulted from the stimulation of monocytes by the cell wall components peptidoglycan and teichoic acid or extracellular toxins with superantigenic properties that stimulated MHC-dependent T cell proliferation (Lina et al. 1996; Goda et al. 2021). Previous studies have reported clinical cases of TSS caused by CoNS species (Crass and Bergdoll 1986; Lina et al. 1996; Goda et al. 2021). However, little is known about TSS caused by CoNS in poultry. Further researches are needed to determine how TSS is induced by the CoNS infection.

According to Szafraniec et al. (2020), *S. chromogens and S. gallinarum* have been identified as etiological factors responsible for systemic infection in broilers, while S*. epidermidis*, *S. hominis*, and *S. saprophyticus* are mainly associated with chondronecrosis with osteomyelitis and septicemia in broilers. *Staphylococcus warneri* has been isolated from broilers with scabby hip syndrome and septicemia (Szafraniec et al. 2020).

### CoNS re-isolation

Overall, it was found that the highest re-isolation rate of CoNS from the challenged groups was from the liver, with a percentage of 71.4%, followed by the intestine and heart, with percentages of 68.6% and 60%, respectively. On the other hand, the yolk and lung had the lowest re-isolation rates of 20% and 28.6%, respectively (Table S4). The highest re-isolation was recorded in groups VII, VII, and V challenged with *S. warneri*, *S. saprophyticus*, and *S. gallinarum.* CoNS in group VII was re-isolated from the liver, heart, intestine, and lung at a percentage of 100% each, while in group VIII it was re-isolated from the liver, heart, and yolk at a percentage of 100% each and from the intestine and lung at a percentage of 80% each. Re-isolation of CONS in group V from the intestine was at a percentage of 100%, while in the other organs it was at a percentage of 20% each (Table S4).

*Staphylococcus* species is one of the major causes involved in the delay of yolk resorption in 1-day-old chicks, which leads to mortality in the first week after hatching (Rehab et al. 2021). In this study, *S*. *gallinarum* and *S*. *saprophyticus* in groups Vand VII were re-isolated from the yolk at a percentage of 20% each. This might explain the observed mortalities in groups V and VIII challenged with *S. gallinarum* and *S. saprophyticus*.

### Histopathological examination

A normal architecture was observed in the negative control group (Ӏ), while noticeable pathological alterations in the heart, liver, and intestine were recorded in the challenged groups (П, Ш, IV, V, VI, VII, and VIII). These pathological changes resembled the classic histopathological alterations linked to *S. aureus* infection (Liu 2009). All classic pathological lesions, such as congestion and the infiltration of inflammatory cells, could be signs of an inflammatory process and toxins in the blood (Akinkunmi et al. 2014). Shokry et al. (2018) revealed classic pathological lesions in the liver and intestine in 10-day-old broiler chicks infected with *S. xylosus*, *S. sciuri*, and *S. lentus*. Stępień-Pyśniak et al. (2017) have reported severe endocarditis associated with *S. simulans* infection in broilers.

**Fig. 2:**
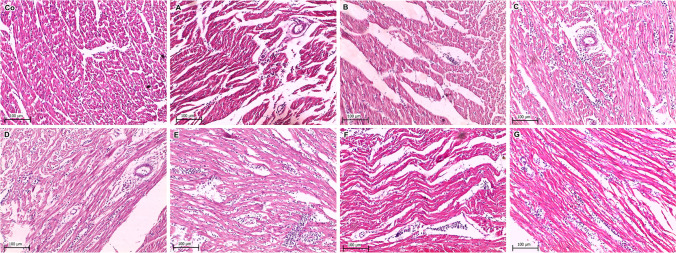
Histopathological lesions in the heart collected from the negative control and experimentally inoculated broiler chicks. **Co** Normal heart tissue, **A** mild myocardial edema and lymphocytic and heterophilic cells infiltration, **B** moderate myocardial edema infiltrated with lymphocytic cells infiltration, **C** moderate myocarditis with severe diffuse leukocytic cells infiltration and perivascular edema, **D** diffuse myocardial necrosis infiltrated with massive leukocytic cells, **E** severe myocardial necrosis replaced by mononuclear cells infiltration, **F** moderate myocardial edema with lymphocytic cells infiltration, **G** mild myocarditis with diffuse lymphocytic and cells infiltration mixed with few heterophils (H&E stain)

**Fig. 3:**
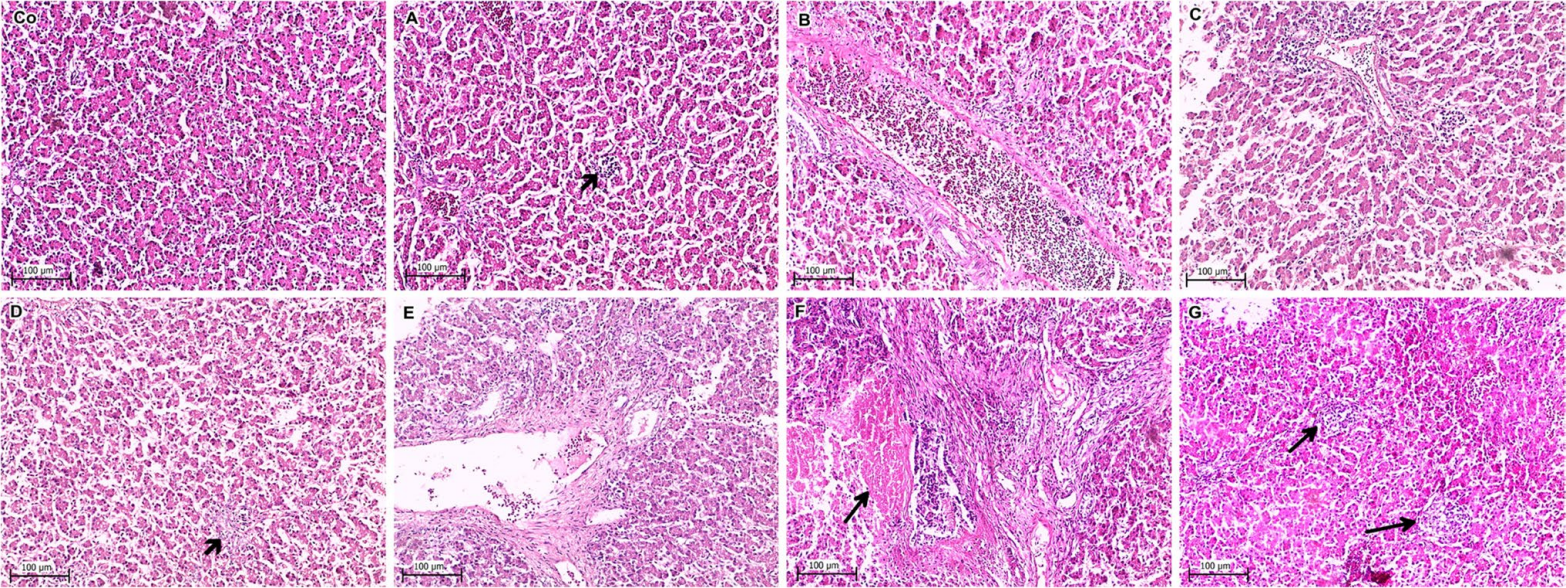
Histopathological lesions in liver collected from the negative control and experimentally inoculated broiler chicks. **Co** Normal hepatic tissue, **A** mild hepatitis with congested sinusoids and focal lymphocytic cells infiltration, **B** severe dilatation of central vein surrounded by degenerated hepatocytes, **C** mild hepatitis with congested sinusoids and perivascular lymphocytic cells infiltration, **D** focal necrotic hepatitis (arrow) with sinusoidal dilatation, **E** diffuse necrotic hepatitis with obvious lymphocytic cells infiltration, **F** severe necrotic hepatitis (arrow) with massive lymphocytic cells infiltration, **G** moderate focal necrosis of hepatocytes replaced by lymphocytic cells infiltration (H&E stain)

**Fig. 4:**
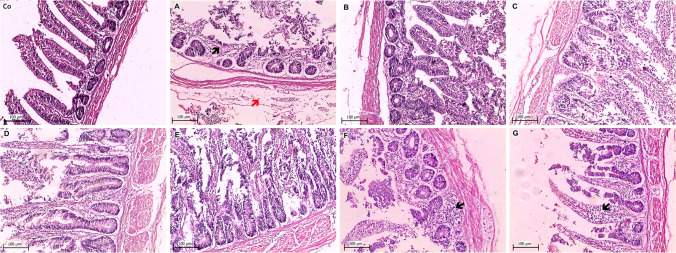
Histopathological lesions in intestine collected from the negative control and experimentally inoculated broiler chicks. **Co** Normal intestinal tissue, **A** severe necrotic enteritis with submucosal edema mixed with lymphocytic cells infiltration (red arrow) and noticeable necrotic villi (black arrow), **B** mild enteritis with activation of intestinal glands and lymphocytic cells infiltration, **C** mild necrotic enteritis with hyperplasia of intestinal glands and submucosal edema, **D** detachment of intestinal villi with lymphocytic cells infiltration and hyperplasia of intestinal glands, **E** moderate enteritis with detachment of intestinal villi and hyperplasia of intestinal glands, **F** severe necrotic enteritis infiltrated with massive lymphocytic cells (arrow), **G** severe mucosal necrosis replaced by massive lymphocytic cells infiltration (arrow) and submucosal edema (H&E stain)

In this study, the heart was the most affected organ, which displayed prominent myocarditis and myocardial edema as well as massive leukocytic cell infiltrations, primarily lymphocytes, heterophils, and few myelocytes that were preceded by myocardial necrosis in groups IV, V, and VI challenged with *S. epidermidis*, *S. gallinarum*, and *S. chromogens* (Fig. 2 and Table S5). The liver showed mild to moderate hepatocellular degeneration and dilatation of hepatic sinusoids and central veins with lymphocytic cell infiltrations. Focal hepatic necrosis was noticed in groups IV, V, VI, VII, and VIII, challenged with *S. epidermidis*, *S. gallinarum*, *S. chromogens*, *S. warneri*, and *S. saprophyticus* (Fig. 3 and Table S5). The intestine was characterized by obvious enteritis in all challenged groups, including intestinal mucosa necrosis, abundant inflammatory cell infiltrations, and submucosal edema (Fig. 4 and Table S5). The observed necrosis in the heart, liver, and intestine in this study may have resulted from the cessation of blood supply by bacterial agents and the degradative action of enzymes on the injured cells (Akinkunmi et al. 2014). It was reported that the severity of pathological lesions produced by CoNS strains was mainly attributed to the number and type of virulence factors expressed (Akinkunmi and Lamikanra 2012). The histopathological findings of this study suggested the significance of inoculation dose 10^8^ in assessing the pathogenic potential of CoNS and the contribution of the *sed* and *mecA* genes in the development of the lesions. Other virulence factors such as hemagglutination, biofilm, capsule, and hemolysin might play a role in the pathogenicity of CoNS strains (Akinkunmi et al. 2014). Further research should be directed to these virulence determinants and their effects on the pathogenicity of CoNS.

## Conclusions

The findings of this study revealed increased numbers of vancomycin-, methicillin-resistant, and enterotoxin-producing CoNS isolates, which emphasizes the need to assess their implications for food safety. Furthermore, infection of broilers with CoNS strains can cause destructive effects on certain organs, including the liver, heart, and intestine, which might be fatal depending on their virulence.

### Supplementary Information

Below is the link to the electronic supplementary material.

Supplementary file1 (TIF 4519 KB)

Supplementary file2 (TIF 2751 KB)

Supplementary file3 (DOCX 17 KB)

Supplementary file4 (DOCX 16 KB)

Supplementary file5 (DOCX 14 KB)

Supplementary file6 (DOCX 17 KB)

Supplementary file7 (DOCX 15 KB)

## Data Availability

The authors confirm that the data supporting the findings of this study are available within the article and its supplementary information.
