# Supplementary figures and images for: Characterization and pathogenicity of multidrug-resistant coagulase-negative Staphylococci isolates in chickens
Source: Int Microbiol. 2023 Apr 13;26(4):989–1000. doi: 10.1007/s10123-023-00354-0 (PMC10622361; doi:10.1007/s10123-023-00354-0)

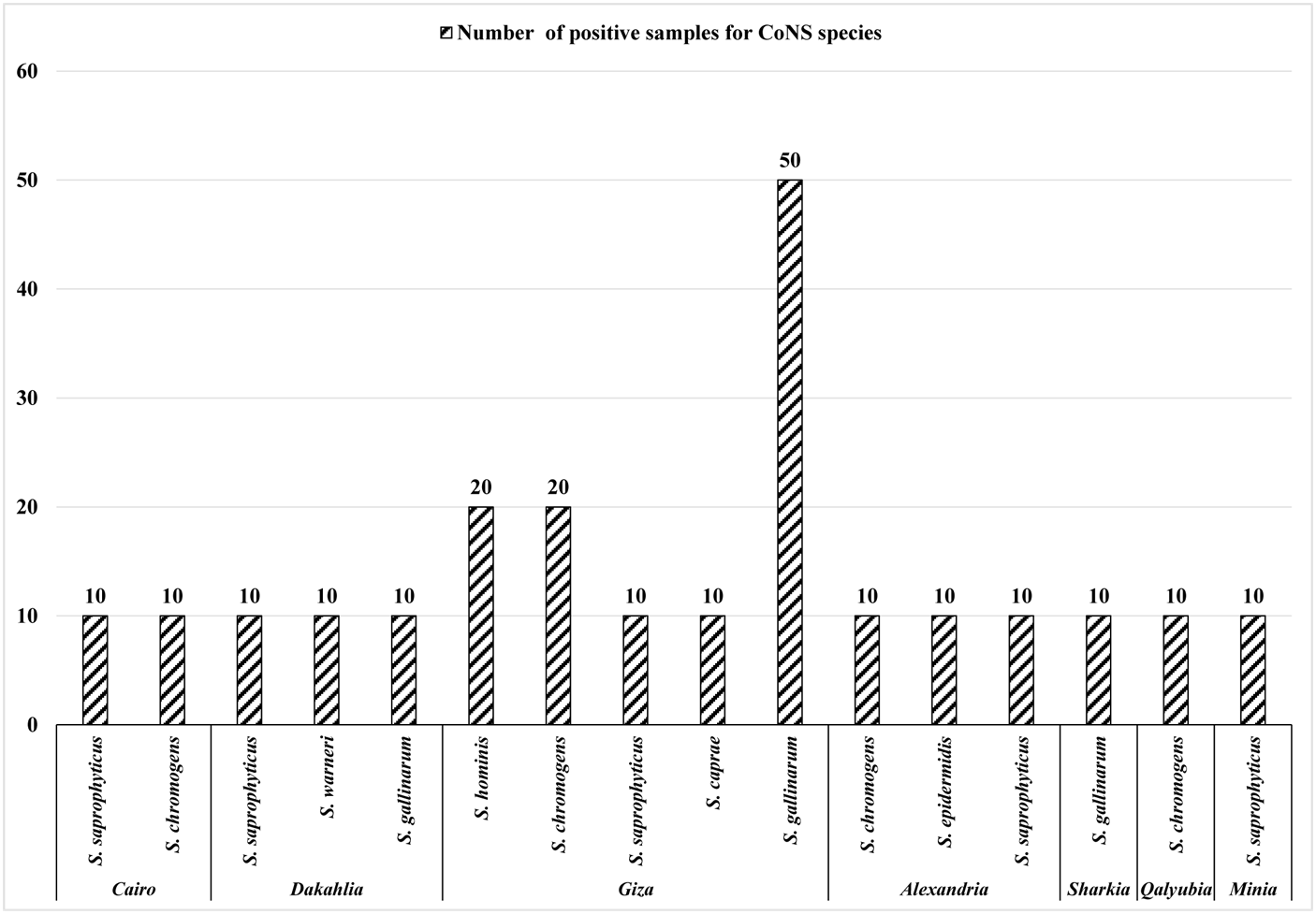

Supplement: Supplementary file 1 — Supplementary file1 (TIF 4519 KB) [file 10123_2023_354_MOESM1_ESM.tif]

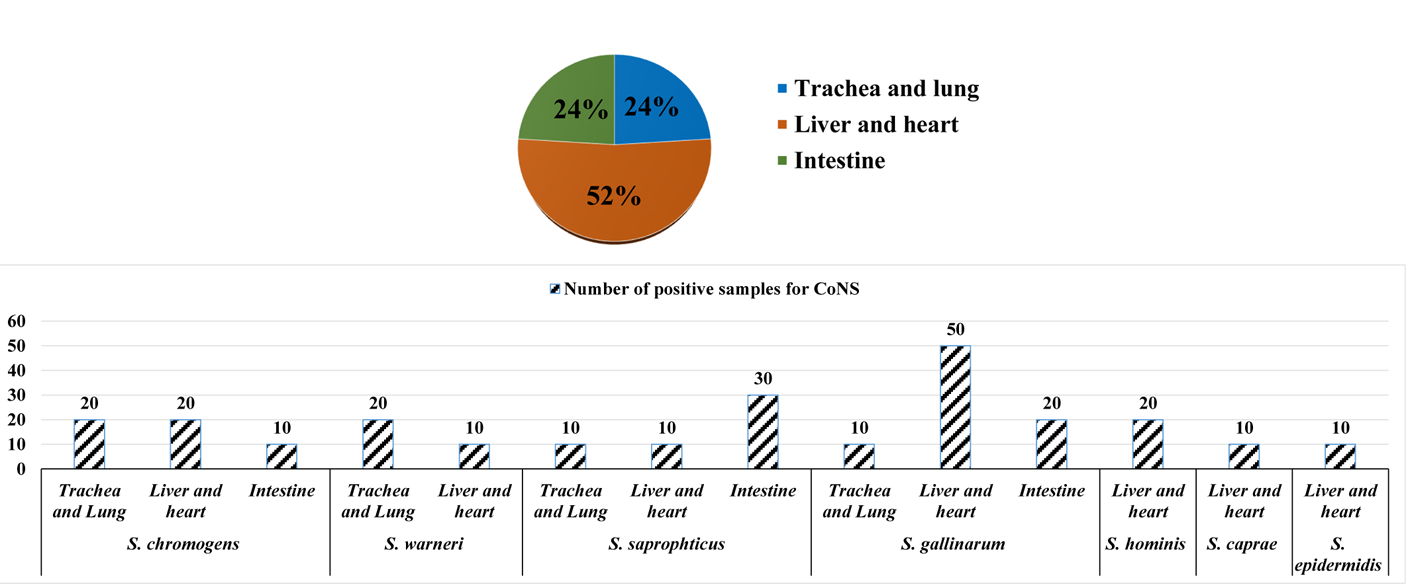

Supplement: Supplementary file 2 — Supplementary file2 (TIF 2751 KB) [file 10123_2023_354_MOESM2_ESM.tif]
