# Supplementary material for: Characterization and pathogenicity of multidrug-resistant coagulase-negative Staphylococci isolates in chickens
Source: Int Microbiol. 2023 Apr 13;26(4):989–1000. doi: 10.1007/s10123-023-00354-0 (PMC10622361; doi:10.1007/s10123-023-00354-0)
Supplement: Supplementary file 3 — Supplementary file3 (DOCX 17 KB) [file 10123_2023_354_MOESM3_ESM.docx]

**Table S1. Biochemical identification of CoNS species**

| Strains | Sugar fermentation test | | | | | | | ONPG (Beta-galactosidase) | Voges- Proskauer (acetoin production) | Novobiocin Sensitivity test | Urea |
| --- | --- | --- | --- | --- | --- | --- | --- | --- | --- | --- | --- |
|  | **Mannose** | **Sucrose** | **Mannitol** | **Trehalose** | **Maltose** | **Lactose** | **Raffinose** |  |  |  |  |
| *S. hominis* | Neg | Pos | Neg | Pos | Pos | Pos | Neg | Neg | Pos | Sensitive | Pos |
| *S. caprae* | Neg | Neg | D | Pos | Neg | Pos | Neg | Neg | Pos | Sensitive | Pos |
| *S. epidermidis* | Pos | Pos | Neg | Neg | Pos | Pos | Neg | Neg | Pos | Sensitive | Pos |
| *S. chromogens* | Pos | Pos | D | Pos | Pos | Pos | Neg | Neg | Neg | Sensitive | Pos |
| *S. warneri* | Neg | Neg | D | Pos | Neg | Pos | Neg | Neg | Pos | Sensitive | Pos |
| *S. gallinarum* | Pos | Pos | Pos | Pos | Pos | Pos | Pos | Weak pos | Neg | Resistant | Pos |
| *S. saprophyticus* | Neg | Pos | D | Pos | Pos | Pos | Neg | Pos | Pos | Resistant | Pos |
| Coagulase | Negative | | | | | | | | | | |
| Catalase | Positive | | | | | | | | | | |
| Sugar fermentation test | Positive (yellow)  Negative (Violet) | | | | | | | | | | |
| ONPG | Positive (yellow color)  Negative (no change in the color) | | | | | | | | | | |
| Voges- Proskauer | Positive (pink red color on the surface)  Negative (no change in the color) | | | | | | | | | | |
| Urea | Positive (red)  Negative (yellow) | | | | | | | | | | |

Neg: negative, Pos: positive, D: 11-89% of strains are positive
