# Supplementary material for: Characterization and pathogenicity of multidrug-resistant coagulase-negative Staphylococci isolates in chickens
Source: Int Microbiol. 2023 Apr 13;26(4):989–1000. doi: 10.1007/s10123-023-00354-0 (PMC10622361; doi:10.1007/s10123-023-00354-0)
Supplement: Supplementary file 4 — Supplementary file4 (DOCX 16 KB) [file 10123_2023_354_MOESM4_ESM.docx]

**Table S2. Target genes, primers, and amplicon sizes used for PCR detection of *16S rRNA,* *sea*, *seb,* *sec*, *sed, see*, and *mecA* genes in the isolated CoNS species*.***

| Target gene | Primers sequences | Amplified segment (bp) | References |
| --- | --- | --- | --- |
|  |  |  |  |
| *sea* | GGTTATCAATGTGCGGGTGG | 102 | Mehrotra *et al*., 2000 |
|  | CGGCACTTTTTTCTCTTCGG |  |  |
| *seb* | GTATGGTGGTGTAACTGAGC | 164 |  |
|  | CCAAATAGTGACGAGTTAGG |  |  |
| *sec* | AGATGAAGTAGTTGATGTGTATGG | 451 |  |
|  | CACACTTTTAGAATCAACCG |  |  |
| *sed* | CCAATAATAGGAGAAAATAAAAG | 278 |  |
|  | ATTGGTATTTTTTTTCGTTC |  |  |
| *see* | AGGTTTTTTCACAGGTCATCC | 209 |  |
|  | CTTTTTTTTCTTCGGTCAATC |  |  |
| *16srRNA* | CCTATAAGACTGGGATAACTTCGGG | 791 | Mason *et al.,* 2001 |
|  | CTTTGAGTTTCAACCTTGCGGTCG |  |  |
| *mec*A | GTA GAA ATG ACT GAA CGT CCG ATA A | 310 | McClure *et al*., 2006 |
|  | CCA ATT CCA CAT TGT TTC GGT CTA A |  |  |
