# Supplementary material for: Characterization and pathogenicity of multidrug-resistant coagulase-negative Staphylococci isolates in chickens
Source: Int Microbiol. 2023 Apr 13;26(4):989–1000. doi: 10.1007/s10123-023-00354-0 (PMC10622361; doi:10.1007/s10123-023-00354-0)
Supplement: Supplementary file 5 — Supplementary file5 (DOCX 14 KB) [file 10123_2023_354_MOESM5_ESM.docx]

**Table S3. Phenotypic and genotypic characterization of the seven selected CoNS isolates used in the experimental study.**

| Isolates | Phenotypic antibiotic resistance pattern | Genotypic |
| --- | --- | --- |
| *S. hominis* 1 | CD, DO, MET, RIF, TE, P, SXT | *16srRNA, sed, mecA* |
| *S. chromogens* 5 | CD, DO, Met, RIF, TE, P, TOB | *16srRNA, sed, mecA* |
| *S. warneri* 3 | FOX, Lev, CD, DO, Met, RIF, TE, SXT, P | *16srRNA, sed, mecA* |
| *S. caprae* | OF, FOX, Lev, CD, DO, Met, RIF, TE, SXT, P, TOB | *16srRNA, sed, mecA* |
| *S. epidermidis* | OF, FOX, Lev, CD, DO, Met, RIF, TE, SXT, P, TOB | *16srRNA, mecA* |
| *S. saprophticus* 3 | OF, FOX, Lev, CD, DO, Met, RIF, TE, SXT, P, TOB, NV | *16srRNA, sed, mecA* |
| *S. gallinarum* 6 | OF, FOX, Lev, CD, DO, Met, RIF, TE, SXT, P, TOB, NV | *16srRNA, sed, mecA* |

OF **(**Ofloxacin), FOX (Cefoxitin), Lev (Levofloxacin), CD (Clindamycin), DO (Doxycycline), Met (Methicillin), RIF (Rifampicin), TE (Tetracycline), SXT (Trimethoprim-sulphamethoxazole), P (Penicillin), TOB (Tobramycin), and NV (Novobiocin).
