# Supplementary material for: Characterization and pathogenicity of multidrug-resistant coagulase-negative Staphylococci isolates in chickens
Source: Int Microbiol. 2023 Apr 13;26(4):989–1000. doi: 10.1007/s10123-023-00354-0 (PMC10622361; doi:10.1007/s10123-023-00354-0)
Supplement: Supplementary file 6 — Supplementary file6 (DOCX 17 KB) [file 10123_2023_354_MOESM6_ESM.docx]

**Table S4. Mortality, re-isolation, and gross lesions in the negative control group and experimental groups inoculated with CoNS species.**

| Groups | Mortality | | Re- isolation | | | | | Gross lesions |
| --- | --- | --- | --- | --- | --- | --- | --- | --- |
|  | **Number**  **(%)** | **Age** | **yolk** | **intestine** | **Heart** | **liver** | **Lung** |  |
| Ӏ  Negative control | 0 | | 0/30 | 0/30 | 0/30 | 0/30 | 0/30 | normal organs |
| П  *S. hominis* | 0 | | 0/30 | 0/30 | 30/30 | 0/30 | 0/30 | polyserositis |
| Ш  *S. caprae* | 0 | | 30/30 | 0/30 | 0/30 | 30/30 | 0/30 | polyserositis, hemorrhage and spotted liver, airsacculitis, yolksac retention  yolk sacculitis 40% (12/30) |
| IV  *S. epidermidis* | 0 | | 0/30 | 30/30 | 30/30 | 0/30 | 0/30 | polyserositis and necrotic heart and liver |
| V  *S. gallinarum* | 6/30 (20%) | 4 day | 6/30 | 6/30 | 6/30 | 6/30 | 6/30 | polyserositis, yolk sac retention and necrotic heart and liver |
|  |  |  | 0/30 | 24/30 | 0/30 | 24/30 | 0/30 |  |
| VI  *S. chromogens* | 0 | | 0/30 | 30/30 | 0/30 | 30/30 | 0/30 | polyserositis, yolk sac retention and necrotic heart and liver |
| VII  *S. warneri* | 0 | | 0/30 | 30/30 | 30/30 | 30/30 | 30/30 | polyserositis and necrotic liver |
| VIII  *S. saprophyticus* | 24(80%) | 2 day | 0/30 | 24/30 | 24/30 | 24/30 | 24/30 | polyserositis, yolk sacculitis yolk sac retention 20% (6/30), airsacculitis and necrotic liver |
|  | 6(20%) | 3 day | 6/30 | 0/30 | 6/30 | 6/30 | 0/30 |  |
| Re-isolation number (%) |  |  | 42/210  (20%) | 144/210  (68.6%) | 126/210  (60%) | 150/210  (71.4%) | 60/210  (28.6%) |  |
