# Supplementary material for: Characterization and pathogenicity of multidrug-resistant coagulase-negative Staphylococci isolates in chickens
Source: Int Microbiol. 2023 Apr 13;26(4):989–1000. doi: 10.1007/s10123-023-00354-0 (PMC10622361; doi:10.1007/s10123-023-00354-0)
Supplement: Supplementary file 7 — Supplementary file7 (DOCX 15 KB) [file 10123_2023_354_MOESM7_ESM.docx]

**Table S5. Histopathological lesions in heart, liver, and intestine collected from the experimental groups inoculated with CoNS species.**

| **Organ** | **Pathological lesions** | **Group П**  ***S. hominis*** | **Group Ш**  ***S. caprae*** | **Group IV**  ***S. epidermidis*** | **Group V**  ***S. gallinarum*** | **Group VI**  ***S. chromogens*** | **Group VП**  ***S. warneri*** | **Group VШ**  ***S. saprophyticus*** |
| --- | --- | --- | --- | --- | --- | --- | --- | --- |
| Heart | Edema | 1 | 2 | 2 | 3 | 3 | 2 | 3 |
|  | Leukocytic cells infiltration | 2 | 2 | 3 | 3 | 3 | 2 | 3 |
|  | Necrosis | 0 | 0 | 1 | 1 | 2 | 0 | 0 |
| Liver | Congestion of central vein | 1 | 3 | 2 | 2 | 2 | 3 | 2 |
|  | Dilatation of sinusoids | 2 | 2 | 2 | 3 | 3 | 3 | 2 |
|  | Degeneration | 2 | 2 | 3 | 3 | 3 | 3 | 2 |
|  | Leukocytic cells infiltration | 1 | 2 | 3 | 2 | 3 | 3 | 2 |
|  | Necrosis | 0 | 0 | 1 | 2 | 2 | 3 | 2 |
| Intestine | Lymphocytic cells infiltration | 3 | 2 | 2 | 2 | 2 | 3 | 2 |
|  | Necrosis | 3 | 1 | 2 | 1 | 1 | 3 | 2 |
|  | Submucosal edema | 3 | 1 | 3 | 1 | 1 | 2 | 2 |

0 = no lesions, 1= mild lesions, 2= moderate lesions and 3= severe lesions
